# Best Basis Selection Method Using Learning Weights for Face Recognition

**DOI:** 10.3390/s131012830

**Published:** 2013-09-25

**Authors:** Wonju Lee, Minkyu Cheon, Chang-Ho Hyun, Mignon Park

**Affiliations:** 1 The School of Electrical and Electronic Engineering, Yonsei University, 134 Shinchon-Dong, Seodaemun-Gu, Seoul 120-749, Korea; E-Mails: delicado@yonsei.ac.kr (W.L.); 1000minkyu@gmail.com (M.C.); mignpark@yonsei.ac.kr (M.P.); 2 The School of Electrical Electronic and Control Engineering, Kongju National University, 275 Budae-Dong, Seobuk-Gu, Cheonan, Chungnam 331-717, Korea

**Keywords:** feature selection, similarity metrics, learning weights

## Abstract

In the face recognition field, principal component analysis is essential to the reduction of the image dimension. In spite of frequent use of this analysis, it is commonly believed that the basis faces with large eigenvalues are chosen as the best subset in the nearest neighbor classifiers. We propose an alternative that can predict the classification error during the training steps and find the useful basis faces for the similarity metrics of the classical pattern algorithms. In addition, we also show the need for the eye-aligned dataset to have the pure face. The experiments using face images verify that our method reduces the negative effect on the misaligned face images and decreases the weights of the useful basis faces in order to improve the classification accuracy.

## 1. Introduction

Pattern recognition algorithms usually use the Lambertian surface, which is covered with the brightness, as the training set. However, this surface cannot be directly applied to the algorithm's classifier since its dimension is too large. Thus, the algorithms generally need compressed images or the use of the Dimension Reduction (DR) stage via image representations such as the Principal Component Analysis (PCA) or Linear Discriminant Analysis (LDA). These techniques can reduce the dimension by leaving only the linearly independent column spaces. This can be shown in the following several methods.

First, Turk's Eigenface uses the face space via the eigenvectors like PCA [1]. This face space is the space projected by using the eigenvectors of the covariance matrix. These eigenvectors are the optimal solution for scattering the training set. That is, they are found via the linear combinations of the principal components that correspond to the directions of the maximal variance in the set. This Eigenface algorithm is of interest and has been researched in recent studies. For example, in 2011 Sudha proposed a principal component neural network with generalized Hebbian learning in order to effectively extract these eigenfaces. In particular, his method can update these eigenfaces when face datasets are changed. As a result, his method improved the accuracy at various light intensities and for various facial expressions [2]. Also, choosing an optimal number of these eigenfaces, dimension of face space, is important in the DR stage. Thus, Liu determined it by considering a proposed relative magnitude of eigenvalues and Meytlis proposed its range via psychophysics experiments [3,4].

Second, the reduction is also needed when classical LDA is applied, since this LDA frequently causes the singular problem of the total scatter matrix. Therefore, Belhumeur proposed the subspace–based LDA, including the DR stage, for this matrix to be the full rank. This can be possible by eliminating the null spaces of the within–class scatter matrix via the use of the DR stage before the classical LDA is applied [5,6]. This is because these null spaces occur when adjacent pixels are similar to each other and thus they are hard to correctly be classified. However, this approach has a significant problem, since Ye discovered that these eliminated null spaces of the within–scatter matrix have important discriminant information [7].

Thus, Yu proposed the direct LDA, which eliminates the null spaces of the between–class scatter matrix instead of those of the within–class scatter matrix [8]. However, this trial is also found to be inadequate because of the distinct differences from the classification constraints of the subspace LDA [9,10]. In addition, sometimes this cannot be applied since the direct LDA uses diagonalization, which requires a nonsingularity of the matrix. To reduce the dimension of a covariance matrix, in 2009 the two-dimensional PCA which prevents singular problems was proposed. This is possible because this method does not need to transform the dimension of training images (N^2^ dimensions) into one dimension [11].

However, even now the classical PCA is used for obtaining a subspace and reducing a data noise because of its simple calculation and high performance. For example, Wang proposed a unified subspace using a 3-dimensional parameter space: the PCA, Bayes, and LDA [12]. He verified to improve a classification accuracy at least 10 percent when the Bayesian analysis in the reduced PCA subspace is used. Additionally, Farrell showed that principal components help covariance–based detectors to be robust against a noise [13]. These results show the classical PCA is currently a useful method for the DR stage. Especially, this PCA is essential to the methods which use a covariance matrix since they frequently cause the singular problem because of high dimensions and smoothed regions of training images. Consequently, this paper proposes a selection method of best principal components in order to improve these PCA and LDA algorithms. That is, this paper proposes an alternative, the best feature selection method, for the DR stage in the PCA and subspace LDA via the use of the proposed cost function, which is related to the classification error of the training set in order to leave these useful null spaces. The proposed method can be thought to be similar to the following three algorithms.

First, this can be thought of as a sub–sampling technique like partitioning, since the best basis features are partitions of the prototype. However, this partitioning method divides the human face into nine equal partitions, and then each partition has a different weight based on psychology and neuropathic psychology. As a result, the weights of the eyes, nose and mouth should be strengthened, while the weights of the cheeks and forehead should be weakened. The recognition rate is increased by giving different weights to the different parts of the face image [14]. However, there are no absolute standards with regard to the partitioning weights. On the other hand, our method is based on the above cost function.

Second, our method is also similar to the approach of AdaBoost, the famous face detection algorithm, since the feature weights are basically calculated and applied [15–17]. For example, Lu and Wang apply the LDA features to AdaBoost in order to improve its accuracy [18]. This method uses the eigenvectors in the DR stage as the weak classifier in AdaBoost. However, this does not comply with the AdaBoost theory, which uses only the weak features for the weak classifiers, while these eigenvectors are too strong. [19,20]. Additionally, it is hard to correctly classify the outliers with AdaBoost since it focuses them by increasing their weights [21,22]. The number of training images for the pattern recognition is also limited. Thus, this pattern detection algorithm, AdaBoost, is not suitable for face recognition since it is based on large numbers of images.

Third, the proposed method is somewhat similar to the WkNN algorithm, since the algorithm inserts the weight to the Nearest Neighbor (NN) classifier. However, these weights, **w**, are calculated according to the distance between the prototype and its NN. Thus, it uses an additional normalizing constant (**w** = 1) in the Lagrange multiplier. This is different from the proposed method, which is calculated based on the NN classification error. In other words, the proposed method improves the NN metrics of the Eigenface and Fisherface methods in the pattern recognition algorithm. That is, our metric learning method attempts to learn the distance metric via the use of the cost function from the two constraints, the positive and negative images. Therefore, the proposed method is robust with regard to outliers or misaligned data since it is based on the cost function, which reduces the classification error. After the weight is learned, our method finds the best subset features among the entire basis faces via these learned weights. Such a selection of the basis faces is the major issue since the needless basis vectors have a considerable effect on the classification error.

This paper is organized as follows: Section 2 describes the state of the art in the field of classical pattern algorithms. Section 3 describes the efficiency and advantages of the best basis selection method using the learning metrics for face recognition. Section 4 presents the technical implementation of the proposed method and validates its efficiency through various experiments. We show the high accuracy of the proposed method by reducing the negative effect of the outliers. Finally, Section 5 provides our research conclusions, weighing the benefits and drawbacks of the proposed method.

## 2. Preliminaries

### 2.1. Need for Pure Face for Classical Pattern Algorithm

**Figure 1.**
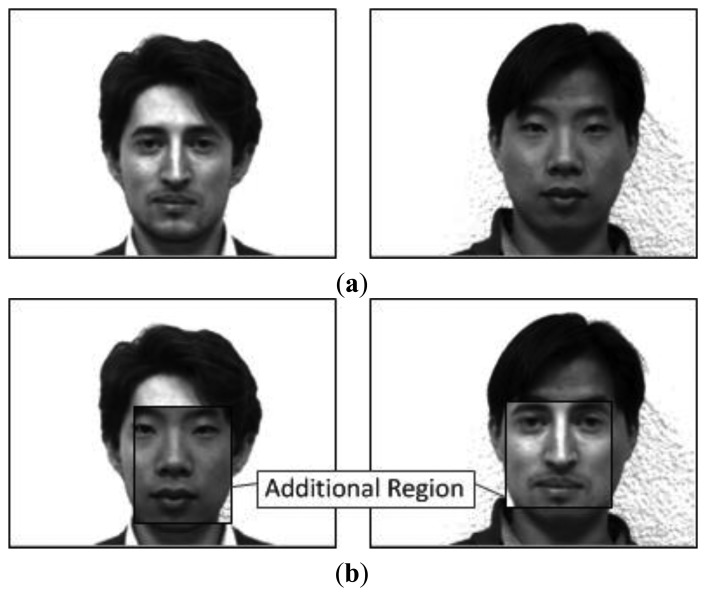
Face images for Chen's experiments: (**a**) Normal faces; (**b**) Mixed faces.

In pattern recognition, the image dataset has a dramatic effect on the classification result. Thus, the famous images were obtained in a limited environment with stationary light and backgrounds. Additionally, these images were measured in a limited region, which includes both the front and side of the face, shoulder, and hair. These limitations are because the researchers believe that the pattern recognition algorithm can find only the important features of a pure face, such as the eyes and nose. However, in 1998 Chen raised questions about this belief and changed the measured region and environment for his experiments [23]. As a result, he knew that these limited images are not suitable for human recognition, because he proved that the discriminant information of the hair and edge regions was more important than those of the pure face by his experiments using the classical pattern recognition algorithms [24]. In other words, these outline images are suitable for face detection rather than face recognition because of the high differential edge information. Figure 1 shows the face images for Chen's experiments. This experiment used these mixed images in which the man's hair is changed, and then Chen tested the problem to determine whether or not the classical pattern recognition algorithm can correctly classify these mixed images. This experiment result shows that the hair features are much stronger than those of the pure face. This means that we must use only the cropped pure face to correctly recognize the face.

To meet this demand, Yale University published a set of cropped pure face images, CroppedYaleB, and these images were also measured in variant lights and expressions for use in various pattern classification experiments [25]. These face images were measured by keeping the eyes aligned to limit the pure face. This is also necessary because the classical pattern recognition algorithm generally uses the mean and covariance of the faces [1,26,27]. That is, this algorithm assumes that these faces have a common mean. These efforts are essential for finding the correct patterns of the pure face except for the edge.

However, we easily find recent studies using these outline facial images instead of pure faces [28,29]. This is because face images of typical benchmark face databases (FERET, CMU PIE datasets) include facial outlines [30,31]. Additionally, most researchers are busy trying to propose new high performance algorithms such as a pyramid decomposition [28]. Thus, we should be reminded of the important of these pure faces.

### 2.2. Relationship between Eigenvector and Eigenvalue in Face Space

The face recognition algorithm commonly uses the high resolution images for the training steps. This is because the algorithm needs clearly discriminant information for each person. Therefore, it is unfortunately inevitable that the small sample size problem occurs since the image resolution is larger than the number of the training images [32]. Especially, this problem happens frequently when the classical pattern algorithm scatters the training images. This is because that the scattering procedure requires the nonsingularity of the covariance matrix of the Eigenface or Fisherface algorithm (the Fisherface is also called as the subspace fisher linear discrimination because of the use of the PCA for reducing this matrix dimension). Thus, a dimensionality reduction stage is required in order to assure this nonsingularity of the face images via the projection by the finite eigenvectors. For example, Turk's DR stage is to convert from the image space to the face space as follows:

(1) p=x×v,

where *T* = {**x**_1_, … , **x***_N_*} is a set of training images, *N* is the number of training images, and **v** is the eigenvectors (basis) of the covariance matrix of **x**. Turk and Pentland call these eigenvectors the eigenfaces, since **p** is the position of **x** in the face space [1]. That is, **f** represents the images projected by using these basis faces. After calculating **p**, Turk's eigenface algorithm compares these projected faces to each other by using the similarity metrics. Consequently, the algorithm helps us to easily understand and calculate the high-resolution images by eliminating the multi-collinearity and by reducing the image resolution. Then these reduced images are used for the similarity metrics for the face comparison (e.g., Euclidean distance).

**Figure 2.**
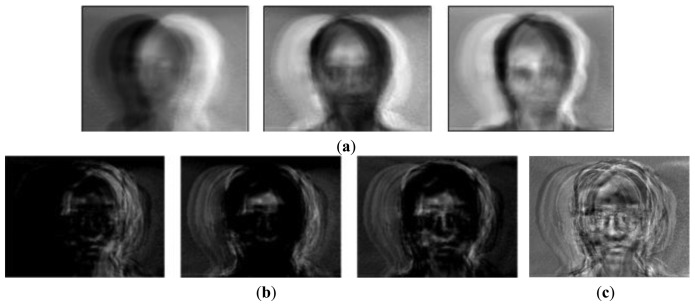
Eigenfaces in descending order of eigenvalue size: (**a**) Eigenfaces (Leftmost: highest eigenvalue); (**b**) Each eigenface–Mean face; (**c**) Mean face.

However, the researchers do not exactly know the relationship between the eigenvalues and eigenvectors in the similarity metrics. That is, it is absolutely normal that the basis faces that have the larger eigenvalues are chosen as the best feature subset [1,27,32] because the classical face recognition algorithms assume that large eigenvalues indicate strong features. However, this is a false belief since Chang's experiments proved that sometimes the first three eigenvectors do not have the discriminant information. Figure 2 shows the eigenfaces in descending order of the eigenvalue size. As expected, the unimportant eigenfaces, including the edges, are shown in the leading group. This is because both the edges and illuminations have high differential values based on the image pixels.

These false impressions unfortunately continue until now. For example, Huang proposed in 2012 an improved principal component regression classification algorithm [29]. This method includes the PCA procedure before a linear regression classification is applied. He used eigenvectors with large eigenvalues as important features. However, first *n* principal components are excluded from the features in order to obtain the robustness against illumination changes. This means that he knew eigenvectors with large eigenvalues do not always be important. However, he used only this rough method (excluding only first *n* principal components) since he does not exactly know which eigenvectors are important. Additionally, most recent studies using the PCA and two-dimensional PCA also regard the eigenvectors with large eigenvalues as the strong features [33–38]. However, as mentioned before, from Chen's experiments we know that the eigenfaces with large eigenvalues do not indicate strong features in face recognition [39]. Consequently, this paper proposes a new selection method of best eigenvectors, eigenfaces for face recognition which is independent to these eigenvalues.

## 3. Best Subset of Basis Faces Using Learning Metrics

As mentioned in the previous section, the classical face recognition algorithms (PCA, LDA) absolutely depend on the basis faces. Thus, this paper proposes similarity metrics for obtaining the best basis subset by adjusting the weights of the basis faces and then selecting only the useful basis faces. For this reason, the proposed method is included in variable selection or feature selection [40–44]. To learn the similarity metrics, our method uses the cost function that minimizes the classification error and the selection criteria that excludes the needless features. It is based on Paredes' distance metrics learning [45]. Thus, each basis face is evaluated differently according to the effects on the classification errors. As a result, it prevents dimensionality problems when the useful basis is not considered [46].

### 3.1. Cost Function Minimizing Classification Errors

The basis faces are not only the features but also results of the eigenface algorithm. That is, the projected face images consist of these basis faces. Thus, it is proper to insert the weights into the projected face images such as **t** or **p***_i_* in order to learn the weights of the basis faces. Suppose the number of training images is *N*, and the number of basis faces is *K*. Then, a weighted distance is as follows:

(2) $$d^{2}(t,p_{i})=\sum_{j}w_{ij}^{2}{\left(t_{j}-p_{ij}\right)}^{2}$$

where *w_ij_* (the *i*-th rows and *j*-th columns), 1 ≤ *i* ≤ *N*, 1 ≤ *j* ≤ *K*, indicate weights of the *i*-th prototype and *j*-th basis face. This similarity method is based on Paredes' NN learning algorithm applied to both a projected prototype, **p***_i_*, and projected test image, **t** [45]. If **w** = 1, all of the basis faces (or prototypes) have same influence on the classification accuracy like the Euclidean distance. If we need the different weights per class, then **w***_j_* = 1/***s****_cj_* where ***s*** is the standard derivation and *c* is the class. These examples show that the weight, **w**, can be used to determine the importance factor of both the prototypes and basis faces.

To learn the weights, **w**, we propose the cost function for minimizing the classification error of the leaving-one-out NN by using the 0–1 loss function as follows:

(3) $$J(w)=\frac{1}{N}\sum_{\text{x}\in T}\text{step}\left(\frac{d(\text{x},x^{=})-d(x,x^{\neq })}{d(x,x^{=})+d(x,x^{\neq })}\right)$$

where **x**^=^ and **x**^≠^ are the NNs that belong to the same class and different class as **x**, respectively. This cost function *J*(**w**) is defined as the summation of the classification errors of all NNs and is based on the following three techniques. The first technique is to define the two NNs as follows. Let **x**^=^ be the **x**'s NN, which is limited in the same class as **x**. Therefore, the distance between **x** and **x**^=^ can be indicated as *d*(**x**, **x**^=^). In the same way, let **x**^≠^ be the **x**'s NN that is included in the class of which is different from that of **x**. As a result, *d*(**x**, **x**^≠^) can be indicated as the distance between **x** and **x**^≠^.

The second technique is to define the classification error as follows. As the definition of the 0–1 loss function, the classification error (1 loss) occurs when the argument of the step function is larger than 0. On the other hand, when its argument is smaller than 1, the classification result is regarded as a 0 loss. That is, the step function in Equation (3) can be used to determine whether or not a classification error happened. This is because the argument of the step function is determined as *d*(**x**, **x**^=^) – *d*(**x**, **x**^≠^). For example, *d*(**x**, **x**^=^) > *d*(**x**, **x**^≠^) means that the classification result is incorrect. Thus, the learning metrics try to reduce the weight of **x**^=^ in order to decrease the distance between **x** and **x**^=^.

The third technique is to be independent of **x**. As mentioned before, the argument of the step function is the difference between two NN distances. Therefore, this argument significantly depends on the values of the NNs. To resolve this dependency, dividing by *d*(**x**, **x**^=^) + *d*(**x**, **x**^≠^) is necessary. This helps the output of the step function to keep a certain boundary even if **x** is varied. Additionally, the step function can be easily calculated by the effect of this boundary. This is particularly useful when the step function is replaced by the sigmoid function *S_β_*(*z*) = 1/(1 + *e*^−^*^βz^*) in order to make it a continuous equation. This is needed in order to exclude the outliers. This is possible because the output of the sigmoid function can be close to zero if arguments of the sigmoid function is just larger than 2.

### 3.2. Gradient Descent Algorithm for Learning Weights

To minimize *J*, we need the partial derivative of *w_ij_*. This is important because that we can determine which of the optimal algorithms is suitable for our approach. That is:

(4) $$\frac{\partial J}{\partial w_{ij}}=\sum_{x\in T}\frac{4S_{\beta }^{\prime }\;(r(x))\{{\left(x_{j}-x_{j}^{=}\right)}^{2}d(x,x^{\neq })-{\left(x-x_{j}^{\neq }\right)}^{2}d(x,x^{=})\}}{{\left\{d(x,x^{=})+d(x,x^{\neq })\right\}}^{2}}w_{ij}$$

where:

(5) $$r(\text{x})=\frac{d(x,x^{=})-d(x,x^{\neq })}{d(x,x^{=})+d(x,x^{\neq })}.$$

Then, by separating the two terms, Equation (4) can be reformulated as follows:

(6) $$\sum_{\begin{array}{c} \forall \text{x}\in T:\\ \text{index}({\text{x}}^{=})=i \end{array}}S_{\beta }^{\prime }\;(r(x))Q(x)\frac{{\left(x_{j}-x_{j}^{=}\right)}^{2}}{d(x,x^{=})}w_{ij}-\sum_{\begin{array}{c} \forall x\in T:\\ \text{index}(x^{\neq })=i \end{array}}S_{\beta }^{\prime }\;(r(x))Q(x)\frac{{\left(x_{j}-x_{j}^{\neq }\right)}^{2}}{d(x,x^{\neq })}w_{ij}$$

where:

(7) $$Q(x)=\frac{4d(x,x^{=})d(x,x^{\neq })}{{\left\{d(x,x^{=})+d(x,x^{\neq })\right\}}^{2}}.$$

This reformulation is needed for the easy analysis by separating into the two term (**x**^=^, **x**^≠^). As shown in Equation (6), if we use ∂*J* / ∂*w_ij_* = 0, then the only solution is 0 since all the terms in this equation depend on *w_ij_*. The Lagrange multiplier is also not suitable for solving this equation since it needs the additional condition about **x**. Thus, we realize that the iterative methods (like the gradient descent) are more proper than the above deterministic methods for solving this cost function. As a result, the gradient descent algorithm is proper to apply for updating *w_ij_* in the negative direction as follows:

(8) $$w_{ij}^{\left(k+1\right)}=w_{ij}^{\left(k\right)}-uw_{ij}^{\left(k\right)}\left[\sum_{\begin{array}{c} \forall \text{x}\in T:\\ \text{index}({\text{x}}^{=})=i \end{array}}S_{\beta }^{\prime }\;(r(x))Q(x)\frac{{\left(x_{j}-x_{j}^{=}\right)}^{2}}{d(x,x^{=})}-\sum_{\begin{array}{c} \forall x\in T:\\ \text{index}(x^{\neq })=i \end{array}}S_{\beta }^{\prime }\;(r(x))Q(x)\frac{{\left(x_{j}-x_{j}^{\neq }\right)}^{2}}{d(x,x^{\neq })}\right]$$

where *k* and *u* are the updating step and the amount (speed), respectively. When simulating Equation (8), if *u* is small or *k* is not big enough, then its optimal solution cannot be reached. This is because the gradient descent is terminated when the updating amount is smaller than epsilon, *ε*, or the learning step is exceeded. On the other hand, if *u* is large, then the local and global minima can be passed. Therefore, we suggest the alternative, which saves costs and weights at every step and then chooses the minimum cost and its weights from the storage. This guarantees that the classification error is smaller than the initial cost [45].

### 3.3. Selection Criterion Excluding Needless Features

As shown in Equation (8), the first term related to **x**^=^ reduces its weight, but the second term related to **x**^≠^ increases its weight. These terms directly affect the distances between **x** and its NNs. That is, the proposed method adjusts the weights of only two NNs (**x**^=^, **x**^≠^) per prototype. These learned weights can be also used to select the best basis subset. This selection criterion is as follows:

(9) $$U_{j}(x)=\frac{1}{N}\sum_{\begin{array}{c} \forall x\in T:\\ \text{index}(x^{=})=m,\\ \text{index}(x^{\neq })=i \end{array}}\text{step}\left(\frac{R_{mj}(x,x^{=})}{R_{ij}(x,x^{\neq })}\right)$$

where:

(10) $$R_{mj}(x,x^{=})=\frac{w_{mj}^{2}{\left(x_{j}-x_{j}^{=}\right)}^{2}}{d{\left(x,x^{=}\right)}^{2}},R_{ij}(x,x^{\neq })=\frac{w_{ij}^{2}{\left(x_{j}-x_{j}^{\neq }\right)}^{2}}{d{\left(x,x^{\neq }\right)}^{2}}$$

Thus, *R_mj_* and *R_ij_* are proportional to the distance between *x_j_* and its NN*_j_*. This means that they are applied by only the *j*-th basis face. Thus, the argument of the step function, *R_mj_*(**x**, **x**^=^)/*R_ij_*(**x**, **x**^≠^), can indicate *d*(*x_j_*, *x_j_*^=^)/*d*(*x_j_*, *x_j_*^≠^). We call this method the Relationship of two *R*s (RR-based distance). This argument means that *U* is the classification error (like the cost function *J*) that is related to the use of the *j*-th basis face. For example, if the value of *U* is large, then *d*(*x_j_*, *x_j_*^=^) > *d*(*x_j_*, *x_j_*^≠^). This means that the *j*-th basis face cannot be trusted and then if we apply this, the classification error is increased. Thus, we need to exclude the *j*-th basis faces from the best subset.

## 4. Experiments and Results

### 4.1. Implementation

Our method was implemented by dividing it into three parts: the acquisition from the image dataset, the conversion into the images projected by the Eigenface, and the application of the learning weights using the gradient descent algorithm.

First, the acquisition is implemented as follows: detailed dataset specifications for all the experiments are shown in Table 1. This part needs the accessing technique for loading the images in sequential order. PainCrop, the image dataset for the first experiment, was used in order to recognize human expressions [47]. As a result, since the number of this dataset is too small, we added additional horizontally symmetric images into the dataset. CroppedYaleB, the image dataset for the second experiment, was used in order to test the effect on the direction and intensity of the light [48]. Then all the images and classes were saved into **x** and **c**, respectively. For example, CroppedYaleB's resolution was 192 × 168. The number of classes and the number of images per class are 17 and 18, respectively. This is a part of the entire images because of the consideration of the computing capacity. For saving the CroppedYaleB dataset, **x** consists of 17 cells, where these cells are equivalent to the classes. Thus, the dimension of the cell was 192 × 168 × 18, since the number of images per class is 18. On the other hand, PainCrop had 12 cells and 14 images per class, so its cell dimension was 241 × 181 × 14. After saving, we converted these 3-dimensional matrixes (cells) into 2-dimensional matrixes since high dimensional matrixes raise the computation load. This is possible because the image dimension was changed to a 1-dimensional matrix. As a result, the cell dimensions of CroppedYaleB and PainCrop were 32,256 × 18 and 43,621 × 14, respectively. Additionally, **c** contained the class of **x**. For example, these values of the first 18 images are all 1 for the first class. The next 18 values are all 2 for the second class. The dimensions of **c** are 1 × 306 and 1 × 168 for CroppedYaleB and PainCrop, respectively. These dimensions are determined by multiplying the number of classes and the number of images per class. After that, these images, **x**, were divided into 10 sets for the 10-fold cross validation, which consists of nine prototype sets and one test set. This is needed in order to improve the experiment's reliability. In addition, the index of these images was only used for reducing the loads that happen when the image data are moved.

Second, the conversion part is implemented in order to obtain the images projected by Eigenface. Thus, we just applied these datasets to the Eigenface algorithm. By using Equation (1), we obtained projected images, where **p***_i_* and **t** are the resulting images from the prototype set and the test set, respectively. Additionally, **c***_p_* and **c***_t_*, which include the true prototype and test classes, respectively, were also obtained in order to use for checking the classification result in the similarity metrics.

Third, the gradient descent algorithm is implemented in order to learn the weights for both the basis faces and prototypes. First, 1 image set among the 10 sets from the 10-fold cross validation is loaded. Next, we needed to know the differences among **x**, **x**^=^ and *index*(**x**^=^) in order to correctly implement this part in Equation (6), because these variables are closely related to each other. Thus, we separate this equation into two individual terms that relate to **x**^=^ and **x**^≠^ for easy implementation.

### 4.2. Experiments for Face Datasets

The following experimental parameters were commonly used for learning the gradient descent algorithm in our experiments. We set these tuning parameters via initial experiments. An updating amount, *u*, was set as a considerably small value (1.0*e*–7) since this affects *J*'s variation which predicts the classification error. That is, this parameter can adjust updating the quantity per step in Equation (8). An epsilon, *ε*, was used for detection when *J*'s derived function is close to zero. That is, our weight updating procedure was finished when the difference between before and after *J* is smaller than this epsilon. Thus, the epsilon was set as a small value (1.0*e*–6) which is close to zero. The slope of the sigmoid function, *β*, was set to 10. If *β* is large, the sigmoid function is similar to the step function. However, it is impossible that the output of the sigmoid function is 0 or 1. In this paper, we used famous datasets that are published by reliable sources. The first experiment used the PainCrop dataset, which is similar to that used in Chen's experiments [23,24].

**Figure 3.**
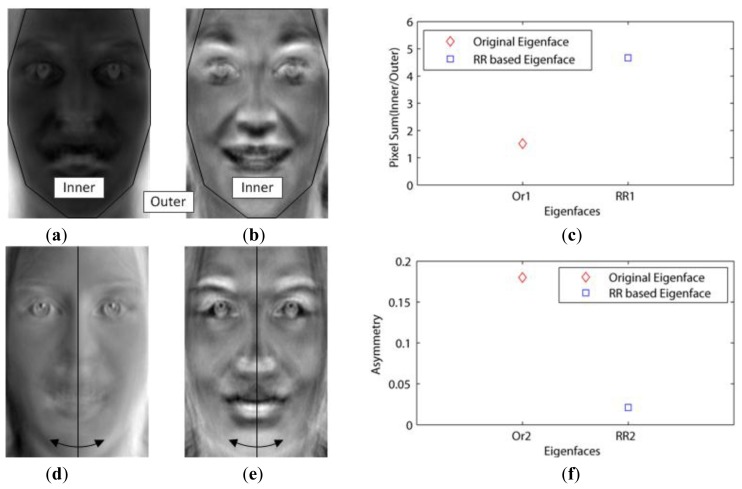
Comparison of eigenfaces chosen by *U* and eigenvalue: (**a**) First basis face with a large eigenvalue (Or1); (**b**) First basis face with a small *U* (RR1) ; (**c**) Pixel summations between inner and outer faces; (**d**) Second basis face with a large eigenvalue (Or2); (**e**) Second basis face with a small *U* (RR2); (**f**) Eigenface asymmetry between Or2 and RR2.

This dataset included not only the facial outlines, but also aligned the faces by the eyes. In addition, this had to be changed into gray images, since it contained red, green and blue images. Then, when using the 10-fold cross validation, we needed to consider whether or not the number of images in the dataset is enough for the validation. If not enough, one of the 10 sets is vacant. This experiment tested for the effect of the basis faces on the classification error. We called Paredes' distance as the weight–based distance in our experiments. Figure 3a,d show the first (Or1) and second (Or2) basis faces that have large eigenvalues (1.0354*e* + 08, 4.9670*e* + 07), respectively.

Figure 3c shows that the Or1 face emphasized the facial outlines or edges as mentioned in Chen's experiments. In this figure, the white pixels represent the important features (basis face) since they are close to 255. This is because these white pixels were emphasized when they were multiplied with the images in Equation (1). On the other hand, Figure 3b,e show the first (RR1) and second (RR2) basis faces that have small values of *U* (0.264, 0.271) via the proposed method, respectively. Figure 3f shows that the important features are related not to the illumination but to the pure faces (RR2), since the eigenfaces (Or2) with high asymmetry values seem to be influenced by the light. This is possible because our method excluded the edge basis faces (outlines) by increasing their weights.

**Figure 4.**
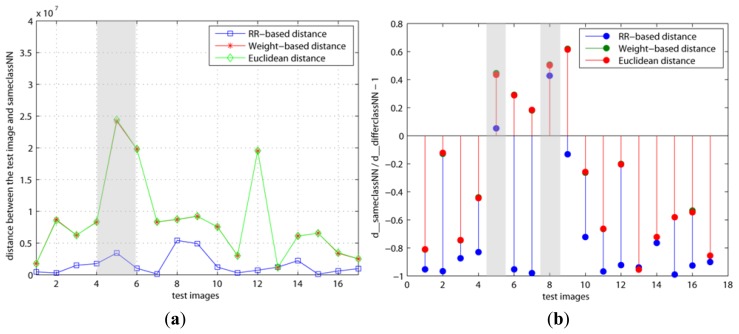
Variation of weights using Euclidean, weighted–based and RR-based distance (*u* = 1.0*e*−7, *ε* = 1.0*e*−6, *β* = 10, *H* = 10, *k* = 5): (**a**) *d*(**x**, **x**^=^); (**b**) *d*(**x**, **x**^=^)/ *d*(**x**, **x**^≠^) − 1.

**Figure 5.**
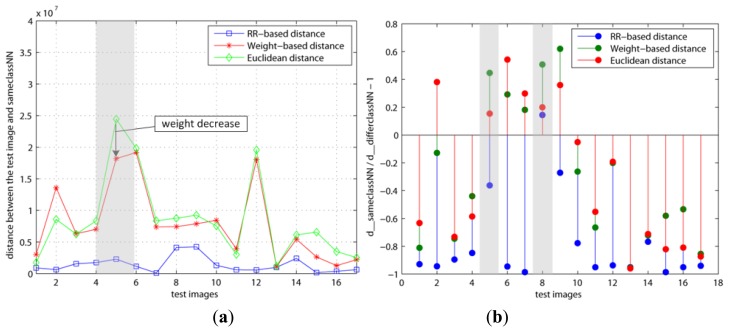
Variation of weights using Euclidean, weighted-based and RR-based distance (*u* = 1.0*e*−7, *ε* = 1.0*e*−6, *β* = 10, *H* = 20, *k* = 200): (**a**) *d*(**x**, **x**^=^); (**b**) *d*(**x**, **x**^=^) / *d*(**x**, **x**^≠^) − 1.

The variations with regard to the weights when we used various basis faces (Or1, Or2, RR1, and RR2) are shown in Figures 4 and 5. As mentioned previously, our method (RR-based distance) chooses the basis faces (RR1, RR2) related to the pure face as the best subset by using these weights. On the contrary, both the original and weight–based distances used the eigenfaces with large eigenvalues such as Or1 and Or2. Figure 4a, b is *d*(**x**, **x**^=^) and *d*(**x**, **x**^=^)/*d*(**x**, **x**^≠^) − 1 when the number of basis faces used is *H* = 10 and the learning step is *k* = 5, respectively. On the other hand, Figure 5 shows the results when *H* and *k* are increased to 20 and 200, respectively. This learning step, *k*, is the maximum repetitive number of the learning procedure. Thus, *k* has to be set as a large value if the updating amount is small value in order for cost function, *J*, to reach its minimum.

Figure 4b shows that the *y* values of both the 5th and 8th test images are larger than 0 in the RR-based distance. This means that these two test images are incorrectly classified since *d*(**x**, **x**^=^) > *d*(**x**, **x**^≠^). On the contrary, Figure 5b shows that the 5th test image's *y* value is smaller than 0 in only the RR–based distance, since the weight of its **x**^=^ is decreased and the added basis faces have a positive effect on it. Additionally, this indicates that the 8th test image needs to apply more basis faces or its weight needs to be updated more. In other words, this shows different results than the other distances since the two other distances applied the eigenfaces with large eigenvalues (Or1, Or2). These differences directly affected the classification error.

**Figure 6.**
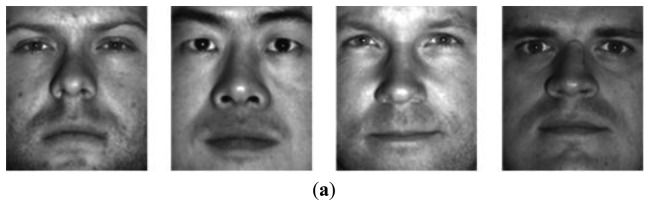

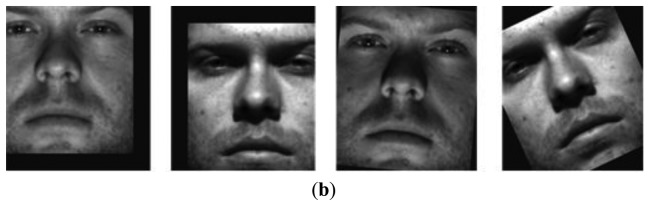
Face images of CroppedYaleB: (**a**) Eye-aligned images; (**b**) Misaligned images.

**Figure 7.**
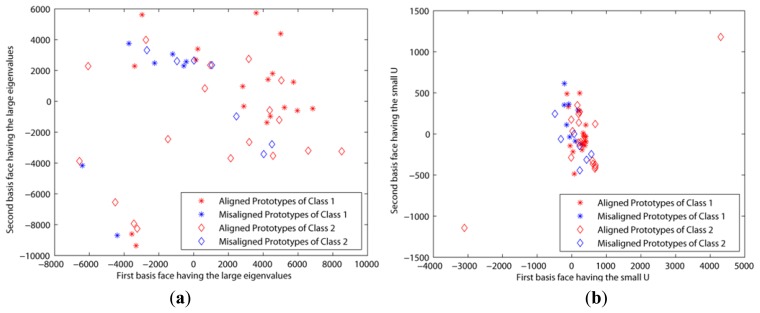
Prototypes projected by two basis faces (*u* = 1.0*e*−7, *ε* = 1.0*e*−6, *β* = 10, *k* = 100, *H* = 10) using CroppedYaleB + misaligned faces: (**a**) Basis faces having the large eigenvalues are applied; (**b**) Basis faces having the small *U* are applied.

**Figure 8.**
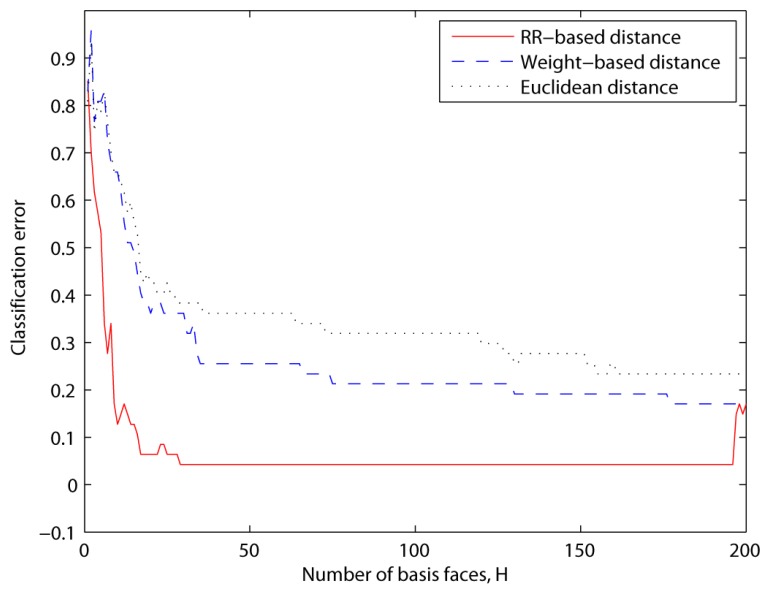
Classification results of Euclidean distances, weight-based distance and RR-based distance (*u* = 1.0*e*−7, *ε* = 1.0*e*−6, *β* = 10, *k* = 100) using subset 2 of CroppedYaleB + misaligned faces.

The second experiment was based on the CroppedYaleB dataset, which was introduced in Section 2. We also added the additional misaligned face images in Figure 6 into this dataset in order to test the robustness to the misalignment. The first misaligned dataset was rotated clockwise and counterclockwise at 5 and 25 degree angles from the aligned dataset. The second misaligned dataset was shifted to the top and bottom 20 pixels from the aligned dataset. Then we needed to crop these moved images to retrieve the original size, 192 × 168, since their sizes were changed by the modifications. Figure 7a,b shows the prototypes per class that are projected by the first two eigenfaces that have large eigenvalues and small *U*s, respectively. That is, these prototypes are scattered by the selected basis faces. In spite of that, the misaligned prototypes were close to the aligned prototypes when the basis subsets with the small *U*s were applied, as shown in Figure 7b. This means that our method chose the basis faces that can correctly classify the misaligned prototypes. On the other hand, the prototypes are scattered in all directions when the other methods using the large eigenvalues were applied. These experiment results directly affect the classification error, as shown in Figure 8. That is, this figure shows that the classification error using the proposed method was close to zero in spite of the misaligned outliers when *H* > 30. In addition, this also shows that the classification error cannot be reduced when we apply the basis faces with large eigenvalues, even if *H* = 300.

**Figure 9.**
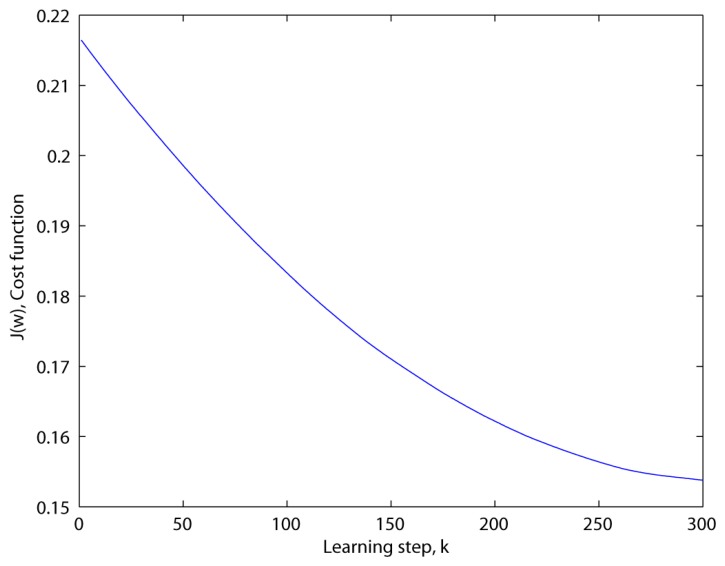
Cost function according to the learning step (*u* = 1.0*e*–7, *ε* = 1.0*e*–6, *β* = 10, *H* = 10).

These results occur because of the following reasons. First, the sigmoid function of the proposed method in Equation (8) can reduce the negative effects on these misaligned prototypes since its slope, *S′*(**x**), is close to 0 when its augment is too small or too large. Second, this method can predict the classification error during the training step by *J* in Equation (3). If the learning weight is correctly calculated, then *J* is a descending curve as shown in Figure 9. This curve shows that our method can choose the basis faces that are suitable for these outliers via the predicted classification error.

The third experiment tested to know differences between the Paredes method and proposed method in Table 2. Thus, both methods used same basis faces for same experimental environment. In addition, same parameters were also used except a learning rate, *u*. Learning rates for the Paredes method and our method are 0.01 and 0.001, respectively. These different values are needed because ranges of two cost functions are different each other.

The fourth experiment shows the classification results using the various datasets, as shown in Table 3. This experiment was tested in order to determine which dataset is proper for use with the proposed method and to compare other distance metrics. For example, the classification result was poor when we tested the Yale Face dataset [49]. This is because this dataset is not aligned with the eyes and includes the face outline, as shown in Figure 2. This is also confirmed by the results of the PainCrop and PainInner datasets (PainInner is the dataset in which the face outline is cropped from PainCrop). That is, our method is more proper for the eye-aligned face images, since the classification error of PainInner is smaller than that of PainCrop. In addition, the experiments were more effective when the additional Horizontally Symmetric Face images (HSF) were inserted into the dataset. For example, we added these HSF images into the Olivetti Research Laboratory (ORL) face database [50], FacE REcognition Technology (FERET) database [51] PainCrop, and PainInner.

The last experiment is the classification results which are compared with two classification algorithms and two principal component selection algorithms, as shown in Table 4. Non-Negative Least Squares classification (NNLS) and Linear Regression Classification (LRC) were used for this classification test [52–54]. In addition, Information Gain (IG) and Sequential Forward Selection (SFS) were also used for this best principal component selection test [55,56]. These algorithms were suitable for our experiment since the performances of these algorithms were recognized by many researchers. However, face images having a lot of vacant space such as the Yale Face and FERET dataset could not be trained several times since the singular problem occurs in calculation. This is because these algorithms do not include the DR stage. That is, this means the classification result was considerably affected by the DR stage. In addition, we knew that the improved DR stage can reduce the possibility of the singular problem.

### 4.3. Discussion

The proposed method is based on Paredes' NN learning algorithm. The main difference with Paredes' algorithm is the application field. That is, Paredes tried to improve the NN classification, but we applied it to find the best subset for the face recognition. This can be confirmed by Equation (1), because the applied **p***_i_* and **t** are the face images projected by the basis faces. This proposed method learns the NN's weights via the cost function in the DR stage. In Equation (3), the cost function *J*, the summation of the output of the sigmoid function about all **x**, is always smaller than 1 since it is divided by the total number of **x**. This means that all of the outputs of the sigmoid function affect this cost *J*. Each output indicates that the wrong classification is when *J* is larger than 1, and the correct result is when it is smaller than 1. In this procedure, the weights of the NNs are updated in reparative steps. As a result, the augment of the sigmoid function is very important. This augment can also identify the outliers by checking whether its value is larger than 2. A large augment value means that the sigmoid function's slope is close to 0. In Equation (8), this indicates that the outliers have little effect on the weights. This advantage was validated in the second experiment.

Various variables were used in this proposed method. It is particularly important that we need to know the exact meanings of **x, x**^=^ and *index*(**x**^=^), since these variables are closely related to each other. Both **x** and **x**^=^ are the prototypes. **x**^=^ indicates the **x**'s NN. *index*(**x**^=^) is the index of **x**^=^. This is also needed in order to know which weights are updated. As mentioned in Section 3, this method does not update all of the weights of the prototypes, but those of the NNs, which are chosen by the similarity metrics in Equation (8). As a result, the weights of some prototypes having the initial weight value of 1 can exist, since the method does not update it. In addition, this method also updates the prototypes that are in the same class with these NNs. For example, if the class of the selected NN is 2, then all of the prototypes including class 2 are updated. This increases the possibility of the correct classification. Additionally, this improves the algorithm speed, since the number of learning weights is reduced by *N*/ the number of images per class.

Updating the weights has to be repeated until the method reaches the minimum of the cost function in Equation (3), as shown in Figure 9. This must be done carefully, since its tight minimum can cause an overfitting problem. This is because this cost function is calculated using the training set rather than the test set. The proposed method provides the learning stop criterion, which consists of *k* and *ε* for finding the cost minimum. Additionally, such gradient descent algorithms have an important problem of choosing the initial condition of the cost function. This is necessary because of the existence of the local minimum. However, in our case it is impossible to change the initial condition, since it is determined by the cost when the learning is begun in Equation (3). Thus, we need to choose the proper training set, as mentioned in Section 2.1.

In the DR stage, we have to decide how our method can reduce an image's dimension. In other words, we decide the maximum number of the best subset, *H*. This is needed because too many features increase the classification errors. In this paper, to solve this problem, we note that each basis face (eigenvector) is independent and orthogonal. Thus, each image can be represented as (Σ basis × projected image). That is, we need to analyze the relationship between the number of combined basis faces and the classification error. This is convex downward, as shown in Figure 8. This is because of the following three reasons: first, in the early stage, the classification error is significantly reduced when *H* < 30. Second, in the middle stage, this relationship function's slope becomes lower since it reaches the limitation or minimum of the classification accuracy. Finally, in the last stage, this curve goes upward since all of the basis faces are included (the needless features are not excluded). Consequently, the maximum number of the best subset, *H*, is chosen when this relationship function is a minimum.

## 5. Conclusions

In this study, we have proposed a best basis selecting method for NN classifiers in the classical recognition algorithms. The primary contribution of the proposed method was to help face recognition algorithms to find correct faces by using only small numbers of basis faces. This improvement was possible because the proposed method provides a simple scheme for learning weights via the cost function related to the classification error and choosing the best subset among the basis faces via these weights. This is validated by our experimental results. That is, they reveal that basis faces with large eigenvalues do not include the high discriminant information in face recognition. This means that important basis faces are not related to the illumination or face outline. In addition, these results also show that face recognition algorithms need to learn proper training images, which are aligned with eyes and are related to the pure face. This is necessary because our method predicts the classification error using these training images via the proposed cost function, and checks whether prototypes are outliers via the sigmoid function of the cost function. However, this is not enough to detect all of the outliers among the training set. In further research efforts, it would be desirable to apply other outlier detecting algorithms to our method.

## Figures and Tables

**Table 1.** Dataset specifications for our experiments.

**Data Set**	**Class**	**Images per Class**	**Total Images**	**Train Images**	**Test Images**	**Dimension of Cell**
Yale Face	15	11	165	149	16	77,760 × 11
CroppedYaleB	17	18	306	276	30	32,256 × 18
CroppedYaleB + Misaligned	17	28	476	429	47	32,256 × 28
Pain Crop + HSF images	12	14	168	152	16	43,621 × 14
Pain Inner + HSF images	12	14	168	152	16	21,463 × 14
ORL face database + HSF images	40	20	800	720	80	10,304 × 20
FERET + HSF images	100	10	1000	667	333	24,576 × 10

**Table 2.** Comparison of Paredes method and proposed RR-based method (*ε* = 1.0*e*−6, *β* = 10, *k* = 100).

**Data Set**		***H***	**Eigenfaces with Large Eigenvalues**		**Eigenfaces with Small *U*s**	

			**Paredes's Cost Function**	**Our Cost Function**	**Paredes's Cost Function**	**Our Cost Function**
CroppedYaleB + Misaligned	1	10	0.6596	**0.6383**	0.1277	0.1064
		20	0.4681	**0.4468**	0.0426	0.0426
		30	0.3191	0.3191	0.0213	0.0213
		40	0.2340	**0.1915**	0.0213	**0.0000**

	2	10	0.6042	**0.5833**	**0.0625**	0.1250
		20	0.4167	**0.3750**	0.0208	**0.0000**
		30	0.2708	**0.2083**	0.0000	0.0000
		40	0.1875	**0.1667**	0.0000	0.0000

Yale Face	4	10	0.3529	0.3529	0.2941	**0.2353**
		30	**0.2941**	0.3529	0.2941	0.2941
		40	0.2941	**0.2353**	0.2353	0.2353

	8	10	0.1875	0.1875	**0.1250**	0.1875
		20	0.1875	**0.1250**	0.1250	**0.0625**
		30	0.1250	**0.0625**	0.1250	**0.0625**
		40	0.0625	0.0625	0.0625	0.0625

**Table 3.** Classification results using various distance metrics (*u* = 1.0*e*−7, *ε* = 1.0*e*−6, *β* = 10, *k* = 100).

**Data Set**		**Error when *H* = 10**			**Error when *H* = 30**			

		**Euclidean Distance**	**Weight-Based Distance**	**RR-Based Distance**	**Euclidean Distance**	**Weight-Based Distance**		**RR-Based Distance**
Yale Face	Subset 1	**0.1875**	0.2500	0.2500	0.1250	0.1250		0.1250
	Subset 2	0.2941	0.2941	**0.2353**	0.2941	**0.2353**		**0.2353**
	Subset 3	0.2353	0.2353	0.2353	0.2941	0.2941		**0.2353**
CroppedYaleB	Subset 1	0.7234	0.7021	**0.1489**	0.3830	0.3404		**0.0638**
	Subset 2	0.6458	0.5833	**0.0833**	0.2917	0.2292		**0.0208**
	Subset 3	0.5417	0.5625	**0.1250**	0.2708	0.2292		**0.0417**
CroppedYaleB + Misaligned	Subset 1	0.6596	0.6596	**0.1277**	0.3830	0.3191		**0.0638**
	Subset 2	0.5833	0.5208	**0.0625**	0.2500	0.1458		**0.0417**
	Subset 3	0.6042	0.6250	**0.1875**	0.3542	0.3333		**0.0625**
Pain Crop + HSF images	Subset 1	0.1875	0.1875	**0.0625**	0.1250	0.1250		**0.0625**
	Subset 2	0.1765	0.2353	**0.0588**	0.0000	0.0000		0.0000
	Subset 3	0.1176	0.1176	**0.0000**	0.0588	0.0588		**0.0000**
Pain Inner + HSF images	Subset 1	0.3750	0.3125	**0.0000**	0.1250	0.0625		**0.0000**
	Subset 2	0.1765	0.1765	**0.0000**	0.0000	0.0000		0.0000
	Subset 3	0.2353	0.1176	**0.0000**	0.1176	0.0000		**0.0000**
ORL face database + HSF images	Subset 1	0.0500	0.0500	**0.0125**	0.0125	0.0125		**0.0000**
	Subset 2	0.0125	0.0125	**0.0000**	0.0000	0.0000		0.0000
	Subset 3	0.0250	0.0250	**0.0125**	0.0375	0.0375		**0.0125**
FERET database+ HSF images	Subset 1	0.3874	0.3874	**0.0811**	0.3874	0.3724	**0.2192**	
	Subset 2	0.3743	0.3743	**0.1018**	0.4102	0.4042	**0.1347**	
	Subset 3	0.3964	0.3964	**0.1321**	0.4264	0.4144	**0.1982**	

**Table 4.** Accuracy comparison of Information Gain, Sequential Forward Selection, RR**-**based distance, Non**-**Negative Least Squares classification, and Linear Regression Classification.

**Data Set**		**IG**	**SFS**	**NNLS**	**LRC**	**RR**
Yale Face	Subset 1	0.2500	0.1875	×	0.1250	0.1250
	Subset 3	0.1765	0.3529	×	0.2352	**0.1176**
	Subset 10	0.1176	0.1176	×	0.1176	**0.0588**
CroppedYaleB + Misaligned	Subset 1	0.2128	0.2979	0.0425	**0.0000**	0.0851
	Subset 4	0.1458	0.1042	0.0833	0.0625	0.0625
	Subset 5	0.1042	0.2292	0.0625	**0.0208**	0.0625
Pain Crop + HSF images	Subset 1	0.2500	0.0000	0.0000	0.1250	0.0000
	Subset 2	0.1176	0.0588	0.0000	0.0000	0.0000
	Subset 7	**0.0000**	0.0588	0.0588	0.0588	0.0588
Pain Inner + HSF images	Subset 4	0.0000	0.0000	0.1176	0.1176	0.0000
	Subset 5	0.1176	0.1176	0.1176	0.1764	0.1176
	Subset 10	0.0000	0.0000	0.1250	0.1875	0.0000
ORL face database + HSF images	Subset 2	0.0000	0.0000	0.0125	0.0125	0.0000
	Subset 6	**0.0000**	0.0125	0.0375	0.0250	0.0125
	Subset 9	**0.0000**	0.0250	0.0250	0.0125	0.0125
FERET database + HSF images	Subset 1	0.2312	×	0.4264	0.4234	**0.0901**
	Subset 2	0.1138	×	0.3682	0.3562	0.1138
	Subset 3	0.2132	×	0.4084	0.4024	**0.1351**
